# Sex‐specific responses to territorial intrusions in a communication network: Evidence from radio‐tagged great tits

**DOI:** 10.1002/ece3.2686

**Published:** 2017-01-13

**Authors:** Lysanne Snijders, Kees van Oers, Marc Naguib

**Affiliations:** ^1^ Behavioural Ecology GroupWageningen University & ResearchWageningenThe Netherlands; ^2^ Netherlands Institute of Ecology (NIOO‐KNAW)WageningenThe Netherlands

**Keywords:** communication network, eavesdropping, great tits, long‐range signaling, sexual selection, social information

## Abstract

Signals play a key role in the ecology and evolution of animal populations, influencing processes such as sexual selection and conflict resolution. In many species, sexually selected signals have a dual function: attracting mates and repelling rivals. Yet, to what extent males and females under natural conditions differentially respond to such signals remains poorly understood, due to a lack of field studies that simultaneously track both sexes. Using a novel spatial tracking system, we tested whether or not the spatial behavior of male and female great tits (*Parus major*) changes in relation to the vocal response of a territorial male neighbor to an intruder. We tracked the spatial behavior of male and female great tits (*N *=* *44), 1 hr before and 1 hr after simulating territory intrusions, employing automatized Encounternet radio‐tracking technology. We recorded the spatial and vocal response of the challenged males and quantified attraction and repulsion of neighboring males and females to the intrusion site. We additionally quantified the direct proximity network of the challenged male. The strength of a male's vocal response to an intruder induced sex‐dependent movements in the neighborhood, via female attraction and male repulsion. Stronger vocal responders were older and in better body condition. The proximity networks of the male vocal responders, including the number of sex‐dependent connections and average time spent with connections, however, did not change directly following the intrusion. The effects on neighbor movements suggest that the strength of a male's vocal response can provide relevant social information to both the males and the females in the neighborhood, resulting in both sexes adjusting their spatial behavior in contrasting ways, while the social proximity network remained stable. This study underlines the importance of “silent” eavesdroppers within communication networks for studying the dual functioning and evolution of sexually selected signals.

## 1 Introduction

Advertisement signaling is usually linked to intersexual selection and intrasexual competition and thus is a key component of a species’ ecology (Berglund, Bisazza, & Pilastro, 1996; Girard, Elias, & Kasumovic, 2015; Goodwin & Podos, 2014; Snijders, van der Eijk, et al., 2015; Waser & Wiley, 1979). Individuals frequently use long‐range signals, signals that propagate beyond territory boundaries and can reach multiple receivers (McGregor & Dabelsteen, 1996), facilitating (Maynard, Ward, Doucet, & Mennill, 2014), maintaining (Garland et al., 2011), or discouraging (Whitney & Krebs, 1975) social associations. Conspicuous long‐range signals are expected to benefit the signaler and the targeted receivers, but they can also form a source of social information to be used by nontargeted receivers, known as eavesdroppers (Danchin, Giraldeau, Valone, & Wagner, 2004; McGregor & Dabelsteen, 1996; Naguib, Fichtel, & Todt, 1999). The use of social information is taxonomically widespread, ranging from invertebrates to fish, frogs, birds, and mammals, including humans (Clément, Wolf, Snijders, Krause, & Kurvers, 2015; Cvikel et al., 2015; Earley & Dugatkin, 2002; Kurvers et al., 2010; Phelps, Rand, & Ryan, 2007; Pope, 2005; Toelch, Bruce, Newson, Richerson, & Reader, 2014). Signal traits relative to an opponent can provide valuable information on the signaler's motivation and quality (Burmeister, Ophir, Ryan, & Wilczynski, 2002; Greenfield, 2015; McGregor & Peake, 2000; Naguib, Kunc, Sprau, Roth, & Amrhein, 2011). Without risking costly physical interactions, eavesdroppers can obtain absolute and relative information on body condition, fighting ability, and age or experience (Davies & Halliday, 1978; Gil & Gahr, 2002; Halperin, Giri, Elliott, & Dunham, 1998) and adjust their behavior accordingly (Naguib, 2005; Oliveira, McGregor, & Latruffe, 1998; Peake, Terry, McGregor, & Dabelsteen, 2002).

In territorial animals, males have been shown to be repelled by male advertisement signals (Krebs, 1977; Nowicki, Searcy, & Hughes, 1998; Sekulic, 1982), while females are expected to be attracted (Amy et al., 2008; Ballentine, Hyman, & Nowicki, 2004; Grafe, 1999; Snedden & Greenfield, 1998) and to use these signals to assess male quality (Bensch & Hasselquist, 1992; Berglund et al., 1996; Byers, Hebets, & Podos, 2010). This dual function of male signaling has however rarely been tested in the same context in one study system. Some of the exceptions are studies on claw waving in male sand fiddler crabs (*Uca pugilator*). Experiments revealed that male crabs changed their claw‐waving behavior in the presence of females, but not in the presence of males (Pope, 2000a). Also, only female receivers responded to a video of a claw‐waving male, while male receivers did not (Pope, 2000b). Similar findings were obtained for display behavior of male wolf spiders (*Schizocosa ocreata*), supporting an intersexual but not an intrasexual function of male signaling behavior in this species (Delaney, Roberts, & Uetz, 2007). Dual functioning of male signaling was shown also in chaffinches (*Fringilla coelebs*), using a combination of a laboratory and a field study (Leitão & Riebel, 2003; Riebel & Slater, 1998). Females in the laboratory as well as males in the wild responded to male chaffinch song, with females preferring long flourishes and males strongly reacting to short flourishes.

Studies on female responses to male signals are usually limited to enclosures or the laboratory, because female attraction in the wild is challenging to observe and to quantify. As a consequence, it remains unknown whether and how signals simultaneously affect males and females in the wild, an insight which is important for the understanding of the dual intersexual and intrasexual functioning of male signaling behavior. Next to this sex bias, studies on responses to signals in the wild are frequently limited to only one or two observed receivers at the time: the resident male (Behr, Knörnschild, & von Helversen, 2009; Naguib & Todt, 1997; Peake, Terry, McGregor, & Dabelsteen, 2001), a single male neighbor (Amy, Sprau, de Goede, & Naguib, 2010; Myrberg & Riggio, 1985; Naguib, Amrhein, & Kunc, 2004), or a single female, usually the mate (Mennill, Ratcliffe, & Boag, 2002; Murphy & Gerhardt, 2002; Otter et al., 1999). To understand the selection pressures that act on sexual signals, it is essential to also consider the response of the wider communication network, consisting of signalers, target receivers, and various eavesdroppers. All the members of the network, including the eavesdroppers, could form an important selection pressure for male signaling behavior when these members change their behavior in response to the signal. Vocal responses of the neighborhood have been documented previously (Fitzsimmons, Foote, Ratcliffe, & Mennill, 2008a), but silent spatial responses on a neighborhood level (male repulsion and female attraction) have yet to be confirmed. To our knowledge, no field study has yet been able to simultaneously track the spatial responses of multiple surrounding male and females when exposed to the same long‐range signal.

**Figure 1:**
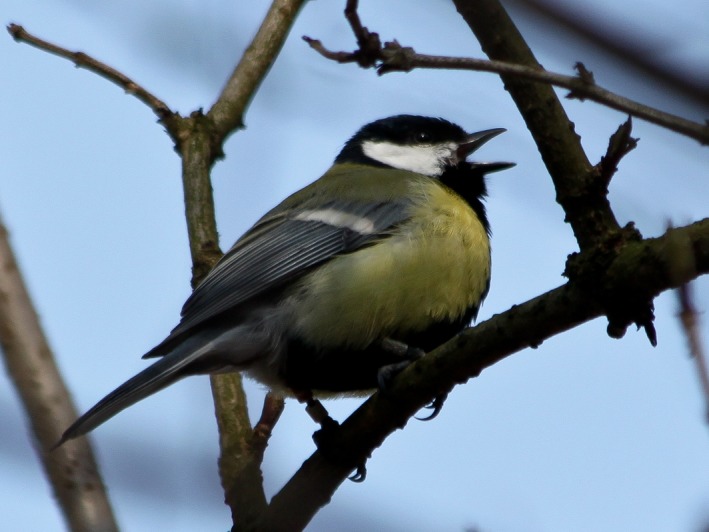
A singing male great tit (*Parus major*)

We here used the novel automatized wireless Encounternet tracking technology (Mennill et al., 2012; Rutz et al., 2012; Snijders et al., 2014) to experimentally study the spatial behavior of male and female great tits (*Parus major*; Figure 1) in response to the vocal interaction between a neighboring resident and a simulated territory intruder. We simulated territory intrusions by playing the song of an unfamiliar male in the territories of male great tits. Subsequently, we monitored the spatial response of the surrounding male and female conspecifics in relation to specific vocal responses of the territory owner. As a strong vocal response commonly indicates that the resident male is in a good condition (Buchanan, Spencer, Goldsmith, & Catchpole, 2003; Gil & Gahr, 2002), we expected sex‐dependent responses with females to be more attracted to stronger vocal responders and males to be more repelled. We investigated three spatial measures in specific: (1) the minimum approach distance of the closest male and female to the intrusion site, (2) the number of male and female connections in the close‐range social network of the vocal responder, and (3) the average duration of these connections. We used these different measures to distinguish between eavesdroppers responding solely to the signal, for example, to sample a potential (extra‐pair) mate from a distance (Otter & Ratcliffe, 2005), and eavesdroppers directly changing their behavior toward the signaler, for example, to approach him in close range. We expected stronger vocal responders to attract neighboring females closer to the intrusion site and to repel neighboring males further away. Also, we expected strong vocal responders to have more and longer close‐range connections with neighboring females, but less and shorter connections with neighboring males.

## 2 Materials and Methods

### 2.1 Study population

We conducted these experiments in March 2014 in a long‐term study population of great tits at Westerheide near Arnhem, The Netherlands (52°01′00.0″N 5°50′20.0″E). Westerheide is a public forest of mixed wood with approximately 200 nest boxes distributed within a 1,000 × 1,200 m area. Using a routine procedure, newly caught birds are tested for exploration behavior using a novel environment test (Dingemanse, Both, Drent, van Oers, & van Noordwijk, 2002; Groothuis & Carere, 2005; Verbeek, Drent, & Wiepkema, 1994). Exploratory behavior, an individual's response to a novel environment (Verbeek et al., 1994), is repeatable and predictive for behavior in many other contexts (Groothuis & Carere, 2005; van Oers & Naguib, 2013). We first tested whether the vocal response of the territorial male was related to his exploration behavior, as shown earlier in our study population (Amy et al., 2010; Snijders, van Rooij, Henskens, van Oers, & Naguib, 2015). See Dingemanse et al. (2002) for details of the standardized novel environment testing procedure.

### 2.2 Spatial tracking

We used the automatic tracking system Encounternet (Encounternet LLC, Portland, OR, USA) to track 44 birds (21 males and 23 females). Encounternet consists of radio transmitters, tags fitted to the birds (Fig. S1), and base nodes, receiver stations, which we distributed in a 40‐m grid across the study site. This triangular 40‐m grid, with mean interbase node distance ± *SD*: 41 ± 6 m, consisted of 166 base nodes positioned in trees at ca. 2.5 m height over an area of approximately 30 ha. This setup allowed us to simultaneously monitor the spatial response of multiple individuals toward territory intrusions in their neighborhood.

On 10 March, we caught all birds that were roosting in the area covered by the base node grid (*N* = 44 birds). The birds were equipped with an Encounternet tag of approximately 1.3 g, using a leg‐looped backpack harness. These tags did not have negative effects on the likelihood of breeding or the apparent survival of the subjects (Snijders et al., 2017). The Encounternet tags are active radio transmitters, set to signal every 5 s. We retrieved the locations of these birds via triangulation, combining all the ID‐coded signal strengths (RSSI) values for each bird within the same half‐minute for at least three base nodes, see (Snijders et al., 2014) and the Supporting information for details.

### 2.3 Simulated territory intrusions

To simulate territory intrusions, we performed playback experiments, broadcasting songs of an unfamiliar male great tit, between 0900 and 1130 a.m. at the nest box a male was roosting in during the tagging procedure. Playbacks were performed from 12 March until 20 March 2014. We performed one to four playbacks on a given day, several territories apart. All playbacks were conducted before the female fertile period; the first egg was laid on 31 March.

We broadcast a great tit recording of approximate two min comprising a repetition of one song type of a great tit recorded at least 4 years earlier (Snijders, van Rooij, et al., 2015). To be able to draw generalizable conclusions and avoid issues with some males potentially hearing the same stimulus multiple times (McGregor, 2000), each subject received songs recorded from a different male. The playback files were constructed using Adobe Audition (Adobe Systems Incorporated, San Jose, CA, USA), by repeating one song of a unique bird with intervals of 4 s for 2 min (Mean ± *SD*: 126.8 ± 15.2 s) or 19 times (Mean ± *SD*: 19.3 ± 1.4) and normalizing the peak amplitude to the same level for all songs. Songs were broadcasted at 79 dB (measured at a neutral site) at 1 m from the loudspeaker with a Voltcraft Digital sound‐level meter 322 (Conrad Electronic SE, Hirschau, Germany). We used a Yamaha NX‐U10 loudspeaker (Yamaha Corp., Hamamatsu, Japan) with a frequency range of 90 Hz–20 kHz, connected with a 25‐m cable to a media player (Archos 405, 30 GB; Archos S.A., Igny, France). The number of stimulus songs played back did not influence any of the quantified subject responses. Also, stimulus file duration did not correlate with the subject response (Table S2).

During the playback, two observers were present. The observers were close to public footpaths, at least 15 m away from the speaker and did not move from their location during the experiment to minimize the effect of their presence. Distances were measured with a Leica RangeMaster 900 (Leica Geosystems AG, Heerbrugg, Switzerland). To determine whether or not the subject was in the vicinity, we used a handheld antenna connected to a laptop computer to detect the subject's signal ID. One observer conducted the playback, while the other observer monitored the signal strength of the subject's radio‐tag. We recorded subject's songs using two Sennheiser ME66/K6 microphones (Sennheiser Electronic GmbH & Co. KG, Wedemark, Germany), with a frequency range of 40 Hz–20 kHz ± 2.5 dB and a Marantz PMD660 solid‐state recorder (D&M Holdings Inc., Kanagawa, Japan), with a sampling frequency of 44.1 kHz and a frequency range of 16 kHz ± 0.5 dB, until two min after the end of the playback. From the recordings and from simultaneously recorded spoken notes, we quantified four vocal response measures: (1) the number of song overlaps by the subject; thus, the number of occasions a subject would not wait with vocalizing until the simulated intruder finished a song, (2) the total number of songs during total observation time (playback + 2 min), (3) the number of songs by the subject during the actual playback, and (4) the total singing duration (s), as well as two spatial response measures: (5) total time spent within 5 min of the loudspeaker (s) and (6) latency to approach within 5 min (s) (Snijders, van Rooij, et al., 2015).

In six of the 21 playback experiments, we neither observed a visual nor a vocal response, nor did we detect the subject nearby during the playback using radio‐tag signal strength. Three of these failed playbacks were repeated, but only during one of these repeats the subject responded. Additionally, in two experiments the responding male was not our radio‐tagged subject, so that also these were excluded from the analysis. In total, we analyzed the response of 14 tagged males. Unfortunately, because of an unintended reset of the tracking system on the final playback day, we could not collect the spatial tracking data for the playback with one of these 14 males. Consequently, the sample size for the subject playback response (*N* = 14) differs from the sample size for the spatial neighborhood response (*N* = 13).

### 2.4 Data analysis

#### 2.4.1 Response to simulated intrusions

We conducted a principal component analysis using Varimax rotation with Kaiser normalization (Field, 2013; Kaiser, 1958) for all six response behaviors (rotation converged in three iterations). The Kaiser–Meyer–Olkin measure of sampling adequacy (Kaiser, 1970) was 0.77, and Bartlett's test of sphericity (Bartlett, 1937) was significant (*p* < .001). Two principal components had an eigenvalue larger than 1. The first component represented the four vocal response behaviors (PC1‐vocal; eigenvalue* *=* *3.6; 59% of total variation explained; Supplementary Table S1), while the second component represented the two spatial response behaviors (PC2‐spatial; eigenvalue* *=* *1.9; 31% of total variation explained; Supplementary Table S1). We used Pearson's correlation tests and *t* tests to examine whether or not age (factor) and condition (continuous) correlated with any of the four original vocal response behaviors (Table S3). We categorized age as second calendar year or older and condition as the residual from a regression of weight over tarsus length.

#### 2.4.2 Changes in spatial structure

To examine effects of territory intrusions on the spatial behavior of nearby conspecifics, we compared the distance to the intrusion site of the closest male and closest female within 60 min before the intrusion to the distance of the closest male and female within 60 min directly after. To examine changes in the close‐range social network of the male vocal responders, following the simulated territory intrusions, we compared the number of unique spatial connection partners, defined as individuals within 10 m in accordance with Snijders et al. (2014), in the 60 min before the intrusion to the 60 min after. Likewise, the average time spent with a connection partner, defined as the total time spent with any connection partner divided by the number of connection partners, in the 60 min before the intrusion was compared to the 60 min after. We hereby distinguished between unique male and female connection partners, “associates” from now on. Mates of the subjects were excluded from the female associates.

Songbirds are known to show long‐term behavioral changes, sometimes up to several days, after a simulated territory intrusion (Akçay et al., 2009; Amrhein & Erne, 2006; Foote, Fitzsimmons, Mennill, & Ratcliffe, 2011; Hall, Illes, & Vehrencamp, 2006; Schmidt, Amrhein, Kunc, & Naguib, 2007). We a priori chose 60 min as a measurement interval to trade‐off effects of increased “random” within‐individual variation with decreasing time intervals and increased time of day effects, consequently comparing late morning to early afternoon, with increasing time intervals. By comparing the spatial behavior of neighbors before the experiment to after the experiment, we aimed to control for differences between neighbors in territory distance to the intrusion site.

#### 2.4.3 Statistical analysis

We used linear mixed‐effect models in the R package “nlme” (Pinheiro, Bates, DebRoy, & Sarkar, 2016) for analyzing the changes in neighbor spatial behavior. The difference in minimum distance to the location of the simulated intrusion, the number of unique associates, and average time spent with associates were the three dependent variables. For each of these three dependent variables, a model was constructed with the interaction of the sex of the associates and the main vocal response as predictor. To account for multiple measures of subject individuals, we used subject ID as random effect. Significance of a potential sex interaction was tested by conducting a likelihood ratio test, comparing the model including the sex interaction to the model excluding the sex interaction. We investigated sex‐dependent effects of the specific vocal response variables when effects of the main vocal response (PC1‐vocal) were *p* < .05. Post hoc testing of the male and female response was conducted with the R package “phia” (De Rosario‐Martinez, 2015), when the interaction of the specific vocal response variable was significant, for example, the number of song overlaps.

Due to an outlier in the models for average time spent with associates (Standardized Residual > 2 and Cooks *D* >  4/(*N* = 13)), evaluated using the R package “influence.ME” (Nieuwenhuis, te Grotenhuis, & Pelzer, 2012), we rank‐transformed the average time spent with associates. The residuals of all the models met the assumptions (Shapiro–Wilk test). The principal component analysis was conducted in IBM SPSS Statistics for Windows, Version 22.0 (IBM Corp., 2013, Armonk, NY, USA). We conducted all other statistical test in R 3.2.1. (R Core Team, 2016, Vienna, Austria) using RStudio Version 0.99.489 (RStudio Inc., Boston, MA, USA). We created the figures using R packages “ggplot2” (Wickham, 2009) and “cowplot” (Wilke, 2016).

## 3 Results

### 3.1 Subject vocal response in relation to individual quality

**Figure 2:**
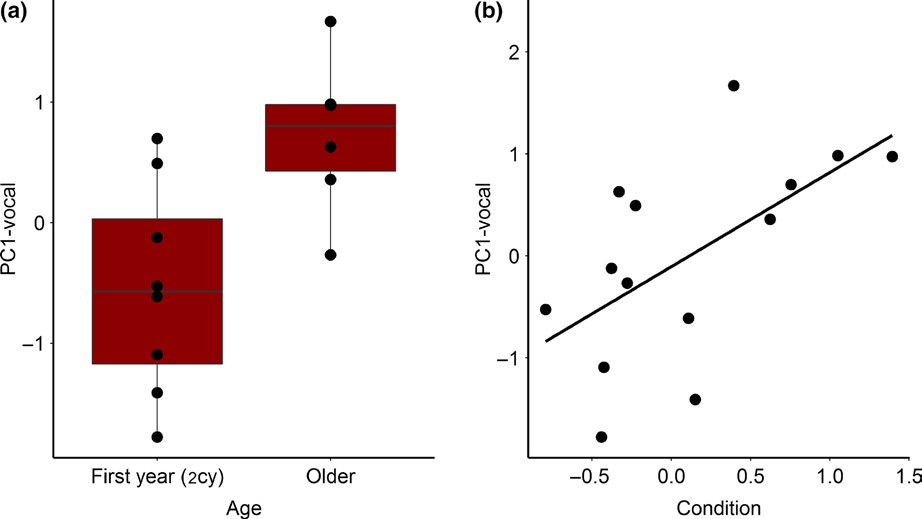
Correlation between vocal response and physical subject characteristics. The main vocal response (PC1‐vocal) of the subjects (*N* = 14) during the playback experiment and 2 min thereafter in relation to (a) age, second calendar year (2cy) or older, and (b) condition, residual of weight over tarsus

Age and body condition were significant predictors of the subject's main vocal response (PC1‐vocal; Welch's two sample *t* test “age”: *t*(11.99)* *=* *−3.10, *p* = .01; Pearson's correlation “condition”: *r* = .59, *N* = 14, *p* = .02; Figure 2). The majority of the separate vocal response variables were stronger for older subjects and for subjects in better condition (Table S3). Moreover, body condition positively predicted the time spent close to the simulated intruder (Pearson's correlation “condition”: *r* = .59, *N* = 14, *p* = .03; Welch's two sample *t* test “age”: *t*(6.7)* *=* *−1.2135, *p* = .27). Older subject males tended to be in better condition (Welch's two sample *t* test “age”: *t*(8.43)* *=* *−1.91, *p* = .09). Exploration behavior did not significantly predict the main vocal response (PC1‐vocal: *r* = −.15, *N* = 14, *p* = .60) or the main spatial response (PC2‐spatial: *r*_S_ = .36, *N* = 14, *p* = .21).

### 3.2 Male and female spatial behavior in relation to subject vocal response

**Figure 3:**
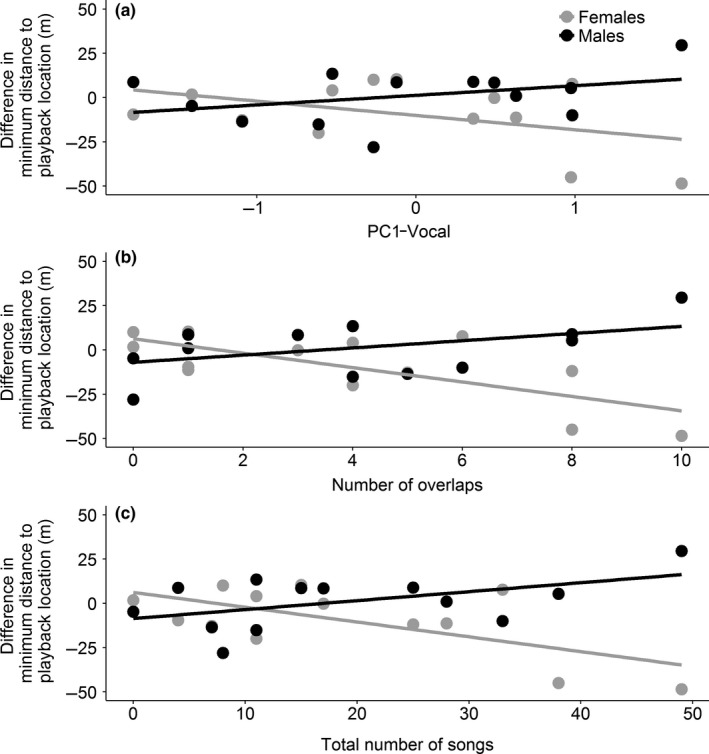
The difference in minimum distance of male and female conspecifics to the intrusion site. The difference between the minimum distance to the playback location before and after the territory intrusion for any closest male (black) or any closest female (gray) (*N* = 13) in relation to (a) the main vocal response of the subject (PC1‐vocal), (b) the number of song overlaps by the subject, and (c) the total number of songs the subject sang. When the difference in minimum distance is negative, conspecifics came closer. Lines visualize sex‐dependent effects

The strength of the male vocal response to the territory intrusion predicted a sex difference in attraction to the intrusion site (sex × PC1‐vocal: Estimate* *=* *13.52, *SE *=* *6.54, χ^2^(1)* *=* *4.61, *p* = .032; Figure 3a). The number of song overlaps, the number of songs sung during the total observation time, and the number of songs sung by the subject male during the playback predicted a decrease in the minimum approach distance of the closest female in contrast to the closest male (Table 1, Figure 3b,c). Females came significantly closer and males tended to stay further away, particularly when subjects overlapped more songs and sang more songs in total (Table 2). These sex‐dependent effects were not directly linked to the age or body condition of the subject (sex × age: Estimate* *=* *13.07, *SE *=* *13.57, χ^2^(1)* *=* *1.07, *p *=* *.30; sex × condition: Estimate* *=* *15.36, *SE *=* *10.53, χ^2^(1)* *=* *2.40, *p* = .12).

**Table 1:** Model comparison statistics for models with the sex × song trait interaction compared to the same model without the interaction

	Estimate	*SE*	χ^2^(1)	*p*
Number of overlaps by the subject	−4.07	1.22		
Sex conspecific	−11.83	13.93		
Number of overlaps by the subject × sex conspecific	6.10	1.72	11.77	**.0006**
Total number of songs	−0.84	0.29		
Sex conspecific	−14.77	9.52		
Total number of songs × sex conspecific	1.34	0.40	10.57	**.001**
Songs during playback	−1.10	0.63		
Sex conspecific	−9.43	11.78		
Songs during playback × sex conspecific	1.80	0.89	4.47	**.03**
Singing duration	−0.10	0.07		
Sex conspecific	−11.83	13.93		
Singing duration × sex conspecific	0.18	0.10	3.67	.06

**Table 2:** Post hoc statistics for significant sex‐dependent effects (*p *<* *.05) of subject vocal response variables on attraction to the intrusion site

	Females			Males		
	Estimate	χ^2^(1)	*p*	Estimate	χ^2^(1)	*p*
Number of overlaps by the subject	−4.07	13.26	**.0003**	2.03	3.29	.07
Total number of songs	−0.84	10.12	**.001**	0.51	3.72	.053
Songs during playback	−1.10	3.65	.06	0.70	1.47	.22

### 3.3 Male and female spatial associations in relation to subject vocal response

There was no overall change in the number of male or female associates (paired Wilcoxon signed‐rank test male associates: *Z* = 0.24, *p* = .81; female associates: *Z* = 1.18, *p* = .22) or in the average time spent with the associates after the territory intrusion (paired Wilcoxon signed‐rank test male associates: *Z* = 0.05, *p* = .96; female associates: *Z* = 0.59, *p* = .55). Changes in the number of female versus male associates (sex × PC1‐vocal: Estimate* *=* *0.22, *SE *=* *0.49, χ^2^(1)* *=* *0.25, *p* = .62) and in the average time associated with female versus male associates (sex × PC1‐vocal: Estimate* *=* *−2.76, *SE *=* *3.14, χ^2^(1)* *=* *0.90, *p* = .34) were not related to the vocal response of the subject.

## 4 Discussion

Female conspecifics reacted differently from males when a neighbor emitted a stronger vocal output in response to a simulated intruder. Where challenged males responded with more songs and overlapped the intruder more often, females came closer to the intrusion site while males stayed away. These findings support our hypothesis that a strong vocal response concurrently fulfills a dual function: attracting females but repelling males. This stronger vocal response to an intruder also positively predicted the challenged male's body condition and age, suggesting that the vocal response to a territory intrusion could be a reliable signal of quality in this species (Grafen, 1990) and thus provide useful information for eavesdropping conspecifics.

Correlations between signal traits and individual quality traits are a common phenomenon in the animal kingdom (Bradbury & Vehrencamp, 2011; Grafen, 1990), but that a long‐range signal of quality simultaneously affects the behavior of both males and females has to our knowledge seldom been verified experimentally in natural circumstances. Relationships between individual quality and signal production do not necessarily mean that receivers are also able to discriminate between these signals and use them for subsequent decision making. How females spatially sample males and which signal traits are used in their decision making is still not well understood (Bensch & Hasselquist, 1992; Otter & Ratcliffe, 2005; Roth, Sprau, Schmidt, Naguib, & Amrhein, 2009). A study on female barking tree frogs (*Hyla gratiosa*) provided some evidence that the females of this species sample males by placing themselves at a distance from a chorus from which they can detect the calls of the chorusing males and subsequently choose a male by approaching him (Murphy & Gerhardt, 2002). The findings in our study may be taken to suggest that territorial female songbirds might sample in a similar way to lekking and chorusing species (McGregor, 2005), by approaching strong vocal performers up to a certain distance, but without necessarily engaging in close‐range contact.

Subject males did not change their number of female associates immediately after the territory intrusion. These findings are interesting as they may suggest that particularly the females that are already within the spatial network of a male are sensitive to his vocal response. It is also possible that females, approaching the intrusion site, initially avoid additional close‐range contact to avoid potential male harassment, aggression of mates (Dale & Slagsvold, 1995), or to first sample other males in the neighborhood. A logical next step for future studies would therefore be to determine whether stronger vocal performers eventually sire more extra‐pair offspring with those females. High‐ranking male black‐capped chickadees were more likely to lose paternity if their mates had heard them lose a singing contest to a simulated intruder (Mennill et al., 2002), and nightingales that responded stronger to playback were more likely to be mated later in the season (Kunc, Amrhein, & Naguib, 2006). Also, female canaries (*Serinus canaria*) gave more copulation solicitation displays to a simulated song overlapping male than to an overlapped male (Amy et al., 2008) and male sac‐winged bats (*Saccopteryx bilineata*) sired more offspring if they were stronger territorial singers (Behr et al., 2006). Yet, male great tits that lost simulated territory intrusions were not more often cuckolded by eavesdropping mates, than males that were allowed to win the vocal interaction (Otter et al., 2001).

The number of song overlaps was an especially strong predictor of the spatial response of neighboring males and females. Also in other animals, including anurans, call overlapping appears to be a source of social information for eavesdroppers on the intention of the sender (Naguib & Mennill, 2010). Competing male gray tree frogs (*Hyla versicolor*) within close proximity vocally overlap each other significantly more than when they are further apart (Reichert & Gerhardt, 2013). Also, there is evidence that in both songbirds and anurans, familiar neighbors try to avoid overlapping each other (Grafe, 2005; Naguib & Mennill, 2010). If vocal overlapping is a signal of aggressive intent (Fitzsimmons, Foote, Ratcliffe, & Mennill, 2008b; Maynard, Ward, Doucet, & Mennill, 2012; Naguib et al., 2004; Naguib & Mennill, 2010; Sprau, Roth, Amrhein, & Naguib, 2012), it is expected to evoke fights that might be too risky for individuals in a lower body condition. Males in lower body condition indeed spent significantly less time close to the simulated intruder in our study. In this way, social retaliation could thus maintain correlations between physical traits and song traits (Anderson, Searcy, Hughes, & Nowicki, 2012; Gil & Gahr, 2002), such as body condition and song overlapping. Whether neighbors were responding specifically to the number of song overlaps by the subject, the total number of songs, or a combination, we cannot disentangle and further research would be necessary. Both vocal response parameters were closely related to the body condition of the subject and thus provided relevant social information to the neighborhood.

Why the spatial repulsion of neighboring males in relation to the strong vocal response of the intruded subject did not translate in corresponding changes to the male‐male close‐range social network remains unclear. These findings could be indicating that social networks among neighboring individuals are robust against disturbances. It is however also possible that strong vocal responders invest more energy in re‐establishing their territory boundaries after an intrusion (Foote et al., 2011; Schmidt et al., 2007), by looking up territory neighbors, while at the same time neighboring males might be avoiding strong responders to avoid potential costly physical interactions. Neighboring great tit males in a previous study moved greater distances after a simulated territory intrusion when their intruded neighbor responded vocally stronger (Amy et al., 2010). If and for what duration close‐range associations will take place after an intrusion is also likely to depend on how familiar neighbors are with each other. Less familiar neighbors might be most likely to receive aggression from the challenged male (Ydenberg, Giraldeau, & Falls, 1988). Follow‐up studies are necessary to reveal the social motivation behind the spatial patterns we observed in this study.

We did not find a relation between exploratory behavior and the vocal response. This is in contrast to three earlier studies that revealed such a relationship, either positively (Amy et al., 2010; Snijders, van Rooij, et al., 2015) or negatively (Jacobs et al., 2014). These varying outcomes between years, populations, and experimental design indicate that personality effects on territorial response are likely to be strongly context specific. However, the sample size in this study was smaller compared to our previous studies so that we cannot rule out that this had an influence on the lack of an exploratory behavior effect.

Taken together, our findings reveal that the vocal response of one territory owner can provide relevant social information on individual quality to the whole neighborhood and that male and female eavesdroppers in the neighborhood adjust their spatial behavior accordingly. When examining the dual function of long‐range signals within the social environment, it is thus of key importance to take the potential selection pressures posed by both male and female eavesdroppers into account. The recent developments in novel tracking technology now provide us with exciting opportunities (Snijders & Naguib, 2017) to further unravel the role of the communication network as selection pressure for sexual signals.

## Conflict of Interest

None declared.

## Data Accessibility

Data are deposited in the Dryad repository: http://dx.doi.org/10.5061/dryad.00v20.

## Supporting information


** **
