# Supplementary material for: Sex‐specific responses to territorial intrusions in a communication network: Evidence from radio‐tagged great tits
Source: Ecol Evol. 2017 Jan 13;7(3):918–27. doi: 10.1002/ece3.2686 (PMC5288255; doi:10.1002/ece3.2686)
Supplement: Supplementary file 1 [file ECE3-7-918-s001.docx]

**Supporting Information**

Sex-specific responses to disturbance in a communication network: evidence from radio-tagged great tits

*Lysanne Snijders, Kees van Oers, Marc Naguib*

**Appendix S1. Extended methodology

Tracking system calibration**

Signal strength detection accuracy was quantified for all the receiver stations (base nodes) by conducting calibration measurements at the end of the tracking period. We approached each receiver up to a two-meter distance (measured with a Leica Rangemaster 900) while carrying an Encounternet tag on a two-meter wooden pole. We adjusted the RSSI values for all receiver stations that deviated more than one standard deviation from the average (based on all 166 receivers) maximum RSSI value (14 ± 4 RSSI). Thus for example, for a receiver, which logged a RSSI of 9 when the test tag was at a 2 m distance, we added +5 to all its RSSI values. Next to these signal strength calibration tests, we also quantified the accuracy of the system in detecting spatial associations. We did this by simulating seven spatial associations throughout the tracking area. During these simulated associations, we moved and turned test tags on two two-meter wooden poles for at least 2 minutes (within 1 m of each other). These tags were located (triangulated) on average 10.8 ± 7.5 m of each other. This is less accurate compared to our calibration tests in 2012, in which test tags were located 7.5 ± 6.5 m apart [[1](#_ENREF_1)]. This discrepancy was possibly caused by the fact that we substantially moved and turned our test tags more in 2014 compared to 2012 (likely more than real birds would have moved and turned). For the actual bird tracking data, in accordance with [[1](#_ENREF_1)], we thus a-priori defined a spatial association as two individuals localized within 10 m of each other (Supplementary Figure S1). Territorial birds within 10 m of each other will likely be aware of each other. Moreover, birds that were actually 20 m or more apart (and possibly unaware of each other) were very unlikely found to be located within 10 m of each other by the system. For example, during calibration tests in December 2012, conducted in the same study area and using a similar grid, test tags that were 20 m or more apart were never located within 10 m of each other (4 transects, each: 20, 25, 30, 50 m).

**Supplementary references**

1. Snijders L., van Rooij E.P., Burt J.M., Hinde C.A., van Oers K., Naguib M. 2014 Social networking in territorial great tits: slow explorers have the least central social network positions. *Anim Behav* **98**, 95-102. (doi:<http://dx.doi.org/10.1016/j.anbehav.2014.09.029)>.

**Table S1**

**Table S1.** Rotated component matrix showing component loadings of the playback response variables. The highest component loading for each playback response behavior is in bold.

| **Playback response variable** | **Component** | |
| --- | --- | --- |
|  | **1** | **2** |
| Number of overlaps | **0.759** | 0.535 |
| Number of songs during playback | **0.924** | 0.302 |
| Total number of songs | **0.939** | 0.114 |
| Singing duration (s) | **0.939** | -0.100 |
| Time spent within 5 m (s) | 0.547 | **0.765** |
| Latency to approach 5 m (s) | 0.087 | **-0.934** |

**Table S2**

**Table S2.** Playback response variables in relation to playback stimulus characteristics. Latency to approach was excluded from this specific analysis given that most approaches happen before the playback of all the songs are finished.

| **Stimulus variable** | **Response variable** | **Test statistic** | **P-value** |
| --- | --- | --- | --- |
| Number of songs | Number of overlaps | *r_s(12)_* = 0.12 | 0.68 |
|  | Number of songs during playback | *r_s(12)_* = 0.18 | 0.55 |
|  | Total number of songs | *r_s(12)_* = 0.10 | 0.74 |
|  | Singing duration (s) | *r_s(12)_* = 0.17 | 0.56 |
|  | Time spent within 5 m (s) | *r_s(12)_* = 0.12 | 0.69 |
| Duration | Number of overlaps | *r_s(12)_* = 0.317 | 0.27 |
|  | Number of songs during playback | *r_s(12)_* = 0.10 | 0.75 |
|  | Total number of songs | *r_s(12)_* = -0.03 | 0.93 |
|  | Singing duration (s) | *r_s(12)_* = -0.10 | 0.74 |
|  | Time spent within 5 m (s) | *r_s(12)_* = 0.39 | 0.16 |

**Table S3**

| **Stimulus variable** | **Response variable** | **Test statistic** | **P-value** |
| --- | --- | --- | --- |
| Condition | Number of overlaps | *r_(12)_* = 0.65 | **0.01** |
|  | Number of songs during playback | *r_(12)_* = 0.71 | **0.005** |
|  | Total number of songs | *r_(12)_* = 0.67 | **0.01** |
|  | Singing duration (s) | *r_(12)_* = 0.51 | 0.06 |
| Age | Number of overlaps | *t_(7.05)_* = -1.36 | 0.21 |
|  | Number of songs during playback | *t_(9.98)_* = -2.84 | **0.02** |
|  | Total number of songs | *t_(8.43)_* = -2.84 | **0.02** |
|  | Singing duration (s) | *t_(11.78)_* = -2.79 | **0.02** |

**Table S3.** Vocal playback response variables in relation to subject body condition and age.

**Figure S1**

**
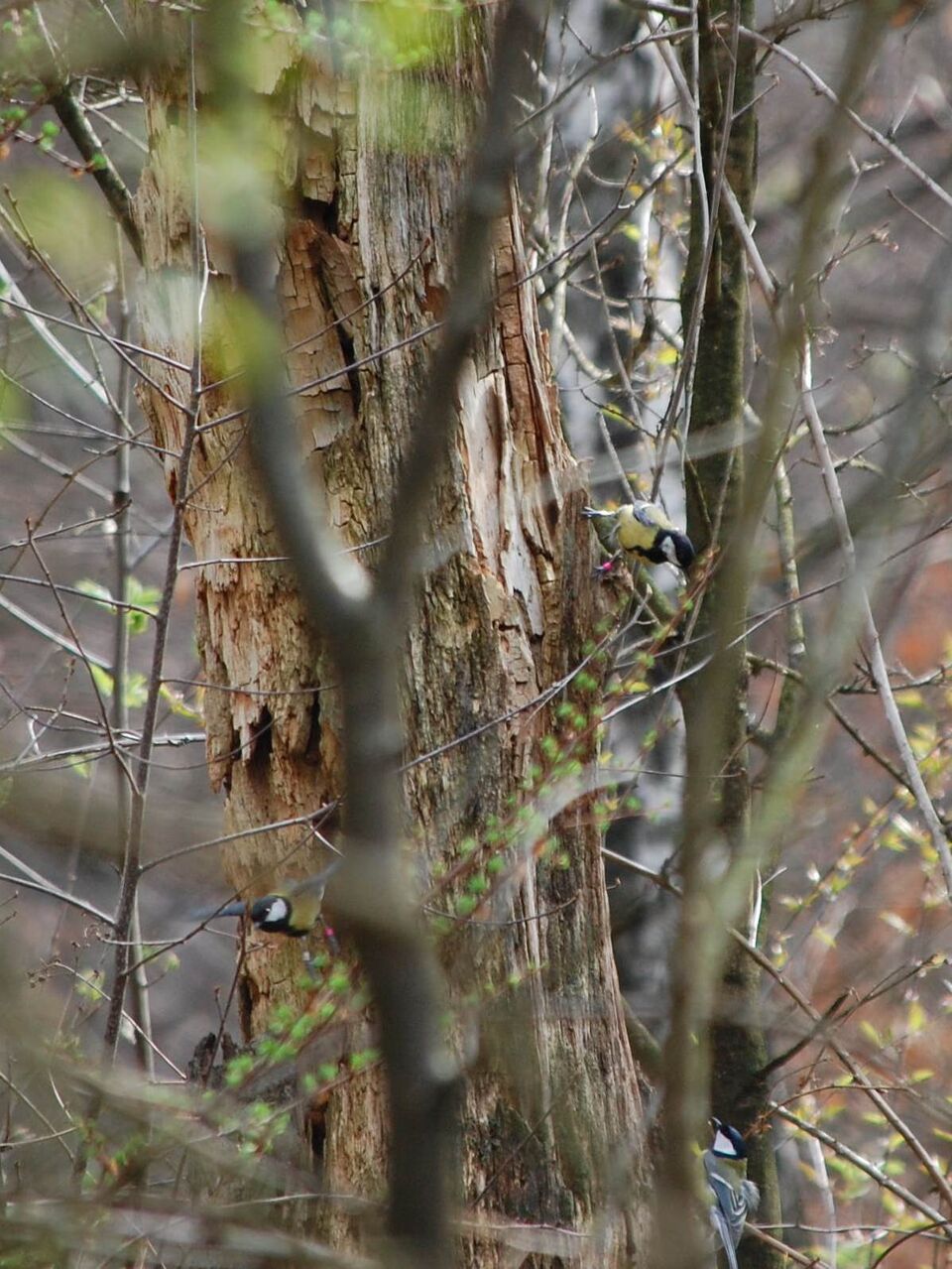
**

**Figure S1.** Three tagged birds, two males and one female, during an encounter.
